# Effect of age and scrotal circumference on sperm morphology in Brahman bulls using a modified fixation technique

**DOI:** 10.3389/fvets.2025.1626425

**Published:** 2025-08-08

**Authors:** Francisco Sevilla, Ignacio Araya-Zúñiga, Arcesio Salamanca-Carreño, Miguel A. Silvestre, Julio Rodríguez, Kenneth Matamoros, Rafael Molina-Montero, Luis C. Carranza-Rojas, Eduardo R. S. Roldan, Anthony Valverde

**Affiliations:** ^1^Doctorado en Ciencias Naturales para el Desarrollo (DOCINADE), Instituto Tecnológico de Costa Rica, Universidad Nacional, Universidad Estatal a Distancia, Alajuela, Costa Rica; ^2^Laboratory of Animal Reproduction, School of Agronomy, Research and Development Center for Sustainable Agriculture in the Humid Tropics, Costa Rica Institute of Technology San Carlos Campus, Alajuela, Costa Rica; ^3^Maestría en Ciencia y Tecnología para la Sostenibilidad, Instituto Tecnológico de Costa Rica, Alajuela, Costa Rica; ^4^Faculty of Veterinary Medicine and y Zootechnics, Cooperative University of Colombia, Villavicencio, Colombia; ^5^Departament de Biologia Cellular, Biologia Funcional i Antropologia Física, Universitat de València, Valencia, Spain, 6 School of Agronomy, Agricultural Production Program (PPA), Costa Rica Institute of Technology, Alajuela, Costa Rica; ^6^School of Agronomy, Agricultural Production Program (PPA), Costa Rica Institute of Technology San Carlos Campus, Alajuela, Costa Rica; ^7^Department of Biodiversity and Evolutionary Biology, Museo Nacional de Ciencias Naturales (CSIC), Madrid, Spain

**Keywords:** reproduction, sperm quality, semen analysis, spermatozoa, Brahman

## Abstract

Sperm morphology (form and size of sperm) analysis is useful for evaluating bull fertility and diagnosing reproductive performance. An advanced age is associated with a higher frequency of morphological sperm anomalies; however, there is limited information on the effect of aging and scrotal circumference on sperm morphological defects in Brahman cattle. The objective of this study was to investigate changes related to age and scrotal circumference on sperm morphology in Brahman bulls in Costa Rica. Brahman bulls are traditionally used in Costa Rican production systems. Sperm morphology was evaluated in 51 Brahman bulls from six age groups (< 24, 24–36, 37–48, 49–60, 61–72, and >72 months old) and scrotal circumference (32–36, 37–41, 42–46, and > 47 cm). The Trumorph^®^ system was used for fixation. A total of 112 ejaculates and 200 sperm cells were analyzed per sample. Sperm defects were classified according to the 2021 World Health Organization laboratory manual and classification of University of Queensland Sperm Morphology Standardization Program. There was a higher frequency of anomalies in younger bulls (< 24 months old) and in those with a scrotal circumference >47 cm. A significant variation was found between the years analyzed, with significant differences (*p* < 0.05) of the year 2022 with respect to the others years. The most common defects by sperm region were loose heads and nuclear vacuoles, distal midpiece reflex, and bent tails. Deterioration related to age and scrotal circumference was observed in sperm morphology, with a higher defect frequency in bulls under 24 months of age and in those with a scrotal circumference >47 cm. Sperm morphology in Brahman bulls showed similar incidence regardless of sexual status (breeding or resting), but they varied according to age and scrotal circumference.

## 1 Introduction

Semen analysis evaluates male infertility (1) by considering various parameters, such as total sperm count, ejaculate volume, sperm concentration, and sperm characteristics (viability, motility, and morphology), as well as composition of ejaculate. These factors significantly contribute to identifying causes of male infertility. Van Leeuwenhoek first reported on sperm variability (2). It was not until the mid-20th century, however, that the relationship between sperm morphological anomalies and subfertility was established (3). In the 1980s, research on male infertility demonstrated a significant decrease in fertilization when fewer than 14% of sampled sperm exhibited normal morphology (4–6).

Criteria for defining sperm as “abnormal” have changed significantly over the past four decades (7). The sixth edition of the WHO manual (8) currently provides the most comprehensive approach to the systematic evaluation of sperm morphology. It has been established that sperm morphology evaluation should be conducted by properly trained personnel familiar with the criteria used to classify sperm cells as abnormal. Moreover, WHO recommends classifying specific anatomical defects (head, midpiece, tail, and cytoplasm) rather than lumping all defects as “abnormal.”

Some studies have found a lack of standardization in semen evaluation methods, including morphology, where different stains are used (9). The University of Queensland has standardized evaluation protocols in its Sperm Morphology Standardization Program (10, 11). The program has developed an impartial approach to sperm morphology assessment. Alternative sperm staining protocols, however, continue to be implemented (9, 12).

The standardization of semen analysis techniques and methodologies ensures that routine procedures, such as sperm morphology assessment, are reliable, robust, and guarantee quality control inter laboratories. Techniques that morphologically evaluate sperm without staining allow for more precise definition of all sperm characteristics (13). The Trumorph^®^ system (Proiser R + D, SL, Paterna, Spain), used in the present study, describes sperm morphology without staining by employing constant pressure and temperature to immobilize sperm (14).

Although there is consensus within the scientific community on what is a “normal” sperm cell, standardization of morphological analysis is limited by the great many methods used to evaluate (15). Traditional protocols typically involve air drying and staining a sample smear, while fewer studies analyze sperm morphology in wet preparations (16). Wet preparations may avoid the traditional dehydration, rehydration, and staining that can alter sperm morphology (17).

Despite the protocol used, sperm morphology is fundamental for evaluating bull fertility (18, 19). For example, it has been correlated with calving rates and embryonic development (20–22), days to conception, and fertility rates in both dairy (23) and beef bulls (24). Fitzpatrick et al. (25) estimated an overall threshold of 30% abnormal sperm to classify a bull as “fit”; however, each type of morphological anomaly requires a specific threshold due to its distinct impact on fertility (11). Additionally, some authors recommend that no more than 20% of sperm cells exhibit head abnormalities (26). Several studies in human and bulls, also show that advanced age is associated with a decline in both sperm motility and the percentage of cells with normal morphology (27–29). Also, in some studies, an effect of the value of scrotal circumference on the percentage of sperm morphology, testicular thermoregulation semen quality and sperm production in cattle has been associated (30).

The Brahman cattle breed is commonly used in Costa Rican production systems (31). Some previous studies on seminal quality in this country have concluded the relevance of the standardization of protocols for andrological analysis, and the variables of seminal quality in productive breeds, including the Brahman breed (32–34). Bull semen quality can be affected by age (35), scrotal circumference (36) and sexual status (33). Older adult bulls have better semen quality than younger bulls (37). Scrotal circumference (SC) is positively associated with semen quality in bulls and closely linked to the age at puberty and fertility in both male and female offspring (38, 39).

In this study, we employed a fixation technique that eliminates the need for staining by leveraging temperature and pressure to effectively immobilize spermatozoa, thereby significantly reducing the risk of artifacts. Thus, the objective of this study was to investigate changes in sperm morphology in beef bulls in Costa Rica related to age and scrotal circumference.

## 2 Materials and methods

### 2.1 Ethical approval

This study was conducted in accordance with the laws and regulations for live animal experimentation in Costa Rica. Throughout the study, animals were handled to ensure their wellbeing and avoid unnecessary stress. The study adhered to ethical research principles, including the three Rs, and was approved by the Committee of the Center for Research and Development of Sustainable Agriculture for the Humid Tropics of the Costa Rica Institute of Technology (CIDASTH-ITCR) in session 08/2023 Article 5.0, DAGSC-075-2023 and CIE-206-2023. The study also followed the ARRIVE guidelines.^1^

### 2.2 Experimental period and study site

This study was conducted from 2020 to 2024 at the La Vega Cattle Farm, located in La Vega de Florencia, San Carlos, Costa Rica (10°25′20.98” N; 84°31′17.57” W) at an altitude of 70 meters above sea level and the La Balsa Farm, which located in San Lorenzo, San Ramón (10°21′8.60” N; 10°31′17.13” W) at 180 meters above sea level. In this area there are two climatic seasons, rainy (from May to October) and dry (from November to April), during the year (39). These farms are affiliated with the Beef Cattle Unit of the Agricultural Production Program (PPA) at the Agronomy School of the Costa Rica Institute of Technology, San Carlos Local Technological Campus. At the time of the study, both farms held a valid Veterinary Operation Certificate, the herd was free of tuberculosis and brucellosis, and health management was maintained through vaccination and deworming programs (CVO-033305-01-F-210-033305). In addition, genealogical and production records were kept for all animals.

### 2.3 Animal management

During the experimental period, the animals were managed in an 18-ha area using a seven-section rotational grazing system, with 6 days of occupation and 42 days of fallow, featuring Ratana grass (*Ischaemum indicum*) and Mombasa grass (*Panicum maximum*). The animals were fed grass without any forage restrictions as well as salt, minerals, and water *ad libitum*. Sanitary bull management included external deworming (e.g., Doramectin), water soluble vitamins (Catosal^®^, Elanco, USA), and mineral supplements (Matsuda breed Top Line, Brasília, Brazil). A general inspection of the bulls was carried out, considering anatomical aspects such as leg and foot shape, body condition, mucous membrane coloration, and any unusual characteristics of each animal. The examination included the condition of the external reproductive organs, including the testicles, scrotum, prepuce, and penis.

### 2.4 Animals and sample collection

Fifty-one Brahman (*Bos indicus*) breeding bulls were selected, ranging in age from 23.4 to 76.2 months old, with a mean of 45.11 ± 18.52 months old (mean ± SD). In 2020 six bulls were used in total; for 2021 13 bulls were used in total; for 2022 10 bulls were analyzed in total; for 2023 15 bulls were analyzed in total; and for 2024 seven bulls were analyzed. The bulls are in a breeding program by natural mating. Bulls had an 84-day mating season (late May to mid-August). Ejaculates were collected prior to and during the mating period. Their body weight ranged from 400 to 850 kg. Age and scrotal circumference were determined at the time of each collection, using farm records and a tape measure, respectively. The average scrotal circumference was 37.42 ± 4.66 cm (mean ± SD), and the mean scrotal temperature was 32.49 ± 1.17°C (mean ± SD). These farms generally retire bulls from the breeding herd at 72 months of age and introduce replacements between 24 and 30 months of age.

Each bull provided at least two ejaculates using the electroejaculation technique described by Ball and Furman (40). The interval between collections was at least 7 days, resulting in a total of 112 ejaculates. An electroejaculator (Pulsator V^®^, Lane Manufacturing Inc., Denver, CO, USA) and a hydraulic press were used to immobilize the animal without sedation. Once the bull was restrained, its general physical condition was evaluated, the movement of the testicles within the scrotal sac was examined, testicular symmetry was noted, and the scrotal circumference (SC) was measured.

The bulls’ ejaculates (*n* = 112) were divided into six age groups [< 24 (*n* = 10), 24–36 (*n* = 28), 37–48 (*n* = 21), 49–60 (*n* = 22), 61–72 (*n* = 22), and > 72 (*n* = 9), months] according to age of the animal at the time of collection. Additionally, they were grouped in four groups by scrotal circumference [32–36 (*n* = 33), 37–41 (*n* = 51), 42–46 (*n* = 17), and > 47 (*n* = 11), cm]. Prior to the experimental period, bulls underwent a sexual rest period of at least 7 days and the maximum of 15 days. They had already passed a standard reproductive fitness evaluation and produced spermatozoa with acceptable characteristics (progressive motility > 60%) and fertility (non-return rates > 65%).

Before electroejaculation, the preputial area was inspected, cleaned, and trimmed to prevent sample contamination. A gloved hand was also inserted rectally to remove any fecal matter from the rectum. Then, a transrectal massage was performed for 1–2 min to stimulate the accessory genital glands (seminal vesicles and ampullae of the vas deferens) and the prostate to relax the anal sphincter prior to introducing the electroejaculator’s probe-electrode. A 75 mm transrectal probe was lubricated and gently introduced into the rectum. The electroejaculator was used in automatic mode, as described by Romano et al. (41). The procedure began with minimal stimuli, then increased according to the bull’s response. By default, 36 cycles of 2 s stimuli were applied automatically. If no response was observed (penis erection) or the bull did not ejaculate within that time frame, the animal was allowed to rest for 5–8 min before initiating another 36-cycle sequence.

### 2.5 Semen collection and processing

Semen was collected directly from the animal’s penis with a sterile disposable collector tube (15 mL Falcon^®^ tube). Subsamples were then placed in Eppendorf^®^ tubes (Sigma Aldrich, St. Louis, MO, USA) containing Optixcell^®^ diluent (IMV Technologies, L’Aigle, France) at a 1:1 (vol:vol) ratio at 37°C. The extender was prepared before collection according to the manufacturer’s instructions, using a 1:2 (vol:vol) dilution with double distilled water.

The samples were placed on a 37°C heating plate while the next sample was collected to prevent thermal shock to sperm cells. Once the collection process was completed, both the Eppendorf^®^ tubes containing the dilutions and the Falcon^®^ tubes with the ejaculates were transported at 37°C in a polystyrene cooler to the Animal Reproduction Laboratory at the Costa Rica Institute of Technology, San Carlos Campus.

### 2.6 Sample preparation for morphological analysis

Sperm samples were prepared from each bull ejaculate. They were gently mixed with a vortex to homogenize them before morphological analysis. From each sample, a 10 μL aliquot of previously diluted semen was placed on a microscope slide and covered with a 22 × 22 mm coverslip. The slide was then introduced into the Trumorph^®^ system (Proiser R + D, SL, Paterna, Spain), which raised the temperature of the sample to 60°C for 20 s and applied a constant force of 196.13 Newtons (N) on the coverslip, as previously described by Soler et al. (14). Once all samples were prepared, they were analyzed using a UB203 microscope (UOP/Proiser R + D) with a 10 × eyepiece and a negative phase contrast 40 × objective to assess sperm morphology. Images were captured using a video camera with a resolution of 768 × 576 pixels. No image retouching was performed. A total of 200 cells were counted per sample, and the abnormality percentage was obtained according to the WHO manual (8) and classification of University of Queensland Sperm Morphology Standardization Program (10, 11). Sperm morphological anomalies are quantified under the premise that the total of all abnormalities plus normal sperm equals 100%. However, coiled tails are excluded, as they may result from handling, and distal cytoplasmic droplets are also excluded, as they may indicate epididymal maturation. Furthermore, numerous distal tails can be observed due to increased ejaculation frequency, which is not strictly considered a defect in sperm production or maturation.

### 2.7 Statistical analysis

Normality and homoscedasticity analyses were performed using the Shapiro–Wilks and Levene tests for sperm morphology. The assumption of normal distribution was verified by using a normal probability plot. Subsequently, an analysis of variance (ANOVA) was conducted to determine the effects of bull age, scrotal circumference, and sexual status, as well as the interactions among these factors. These effects were evaluated in relation to sperm morphology. Additionally, a random residual effect was added to the model to account for correlations among different ejaculates obtained from the same bull. When the afore mentioned effects were significant, mean comparisons were carried out using the Bonferroni test. The results were expressed as mean± standard error of the mean (SEM). Statistical significance was defined as *p* < 0.05. All data were analyzed using IBM SPSS, version 29.0.0.0 for Windows (SPSS Inc., Chicago, IL, USA).

## 3 Results

Descriptive values and mean comparison test of sperm morphological anomalies in bulls by age group were shown in Table 1. The mean of sperm morphological anomalies in bulls that were actively breeding (mating period) was 10.07 ± 1.55%, compared to 12.43 ± 0.95% in bulls at sexual rest. No significant differences (*p* > 0.05) were observed in sperm morphology based on the bull sexual status. Significant differences were observed among the age groups (*p* < 0.05), with a higher incidence of sperm morphological anomalies in young bulls (< 24 months) (17.13 ± 2.01%; CV = 51.87%) except for the age group 61–72 month. Among the other age groups a decreasing trend as age increased. Bulls 24–36 months of age showed the lowest morphological anomaly rate (11.73 ± 1.21%; CV = 28.30%), while the 37–48, 49–60, 51–72, and > 72-month groups exhibited similar values (11.30 ± 1.59%, 10.10% ± 1.86%, 12.51 ± 1.42%, and 10.08 ± 2.32%, respectively; *p* > 0.05). The highest CV was recorded in the 49–60-month group (58.81%), while the lowest was in bulls older than 6 years (2.98%). The results indicate a decline in sperm anomalies with increasing age, and stabilized after 24 months, accompanied by lower variability in older bulls (Table 1).

**Table 1:** Descriptive values and mean comparison test of sperm morphological anomalies in Brahman bulls ejaculates (*n* = 112) by age group from 2020 to 2024 in Costa Rica.

Age (months)	Mean ± SEM	SD	CV	Min	Max	Q3	P (95)
< 24 (*n* = 10)	17.13 ± 2.01^b^	8.88	51.87	12.00	31.50	13.00	31.50
24–36 (*n* = 28)	11.73 ± 1.21^a^	3.03	28.30	2.00	46.50	10.00	20.00
37–48 (*n* = 21)	11.30 ± 1.59^a^	1.07	50.68	2.00	17.00	2.00	2.00
49–60 (*n* = 22)	10.10 ± 1.86^a^	3.77	58.81	1.50	34.50	5.00	14.00
61–72 (*n* = 22)	12.51 ± 1.42^ab^	5.70	48.07	4.50	22.00	14.00	22.00
> 72 (*n* = 9)	10.08 ± 2.32^a^	0.60	2.98	4.00	20.00	20.00	20.00

SEM, Standard error of the mean; CV, coefficient of variation; Min, minimum value; Max, maximum value, Q3, third quartile, P (95): 95th percentile. ^a,b^Different superscripts within a column indicate significant differences (*p* < 0.05).

Descriptive values and mean comparison of sperm morphological anomalies in bulls by SC were shown in Table 2. Significant differences were observed among the SC groups, with higher anomalies in bulls with SC > 47 cm (22.00 ± 4.01%; CV = 43.18%), compared to the 32–36 cm (11.17 ± 1.47%) and 37–41 cm (10.19 ± 1.11%) groups, which showed significantly lower values (*p* < 0.05). The 42–46 cm group had an intermediate value (13.87 ± 2.54%), with no significant differences from other ranges (*p* > 0.05). There was high variability in the data, with CV above 24% in all groups, and the highest values in the 37–41 cm (CV = 39.13%) and > 47 cm ranges (CV = 43.18%) (Table 2).

**Table 2:** Descriptive values and mean comparison of sperm morphological anomalies in Brahman bulls ejaculates (*n* = 112) by scrotal circumference range from 2020 to 2024 in Costa Rica.

SC (cm)	Mean ± SEM	SD	CV	Min	Max	Q3	P (95)
32–36 (*n* = 33)	11.17 ± 1.47^a^	2.63	24.57	4.50	31.50	10.00	20.00
37–41 (*n* = 51)	10.19 ± 1.11^a^	6.40	39.13	2.50	20.00	6.10	20.00
42–46 (*n* = 17)	13.87 ± 2.54^ab^	0.85	41.44	2.00	20.00	5.90	18.00
>47 (*n* = 11)	22.00 ± 4.01^b^	9.50	43.18	5.30	22.00	11.20	22.00

SC, scrotal circumference; SEM, standard error of the mean; CV, coefficient of variation; Min, minimum value; Max, maximum value; Q3: third quartile, P (95): 95th percentile. ^a,b^Different superscripts in the same column indicate significant differences (*p* < 0.05).

**Figure 1:**
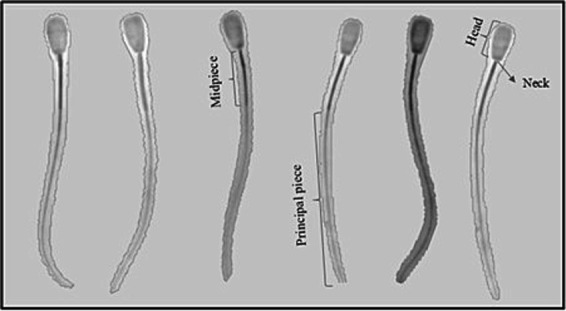
Normal Brahman bulls’ sperm morphology observed using a microscope with phase contrast optics under 400x magnification. Bovine sperm consists of the head, neck, and tail (mid-piece, principal piece and endpiece).

The sperm head exhibited a variety of shapes and sizes, characteristic of a heteromorphic species. A normal bovine sperm morphology consists of the head and tail (midpiece, principal piece and endpiece) (Figure 1).

The mean and standard deviation values were estimated for each of the morphoanomalies identified in the analyzed semen samples (Table 3). In general, the anomalies with the highest mean incidence were bent tails (5.05 ± 5.70%) and nuclear vacuoles (6.04 ± 1.1%), with CVs of 112.89 and 18.21%, respectively. In contrast, the least frequent anomalies were double heads (0.41 ± 0.11%) and broken midpiece (0.34 ± 0.05%), which had CVs of 26.83 and 14.70%, respectively. High variability was observed in anomalies such as proximal cytoplasmic droplets (CV = 102.37%) and tightly coiled tails (CV = 94.76%), indicating a wide dispersion of values within the evaluated population. In contrast, anomalies such as curved midpiece (CV = 10.64%) and teratoid shape (CV = 17.24%) showed less variability. Regarding distribution, the highest 95th percentiles were recorded for curved tails [P (95) = 15.00%] and nuclear vacuoles [P (95) = 7.89%], suggesting that these anomalies reached considerable values in the upper 5% of the individuals evaluated. Analyzing the behavior of morphoanomalies by cell section shows a wide distribution. Seven anomalies were identified in the sperm head, with nuclear vacuoles, loose head, piriform head, and microcephaly being the most common in the analyzed samples (Supplementary Figure 1). On the other hand, six anomalies were identified in the intermediate piece, mostly associated with cytoplasmic droplets and defects in the neck and the intermediate piece (Supplementary Figure 2). Finally, the anomalies at the tail level were mainly related to problems with the natural arrangement of the flagellum (Supplementary Figure 3), which can compromise cell movement.

**Table 3:** Descriptive values and percentages of sperm morphological anomalies in Brahman bulls ejaculates (*n* = 112) during the period 2020–2024 in northern Costa Rica.

Sperm anomalies	Mean	SD	CV	Min	Max	Q1	Q3	P (50)
Sperm head								
Loose head	3.93	2.76	70.33	0.47	18.93	2.00	5.00	3.00
Pyriform head	1.93	1.77	91.35	0.50	10.00	0.50	3.00	1.00
Double head	0.41	0.11	26.83	0.10	1.00	0.30	0.50	0.40
Teratoid	0.58	0.10	17.24	0.40	0.80	0.50	0.67	0.58
Immature form	1.16	0.40	34.42	0.38	2.95	0.90	1.45	1.17
Nuclear vacuoles	6.04	1.1	18.21	1.13	10.20	5.29	6.78	6.03
Microcephaly	1.16	1.09	93.76	0.47	5.00	0.50	1.00	0.50
Sperm midpiece								
Distal midpiece reflex	3.14	1.01	32.16	0.50	6.80	2.44	3.80	3.13
Broken neck	0.40	0.10	25.00	0.02	0.80	0.34	0.48	0.41
Bent midpiece	0.47	0.05	10.64	0.26	0.65	0.44	0.50	0.47
Broken midpiece	0.34	0.05	14,70	0.03	0.69	0.27	0.41	0.34
Proximal droplet	2.04	2.09	102.37	0.47	15.00	1.00	2.00	1.00
Distal droplet	1.35	1.04	48.15	0.10	6.00	1.00	1.90	1.00
Sperm tail								
Tightly coiled tail	2.09	1.98	94.76	0.50	17.00	1.00	3.00	1.00
Coiled tail	2.14	1.72	80.10	0.41	10.00	1.00	3.00	1.50
Bent tail	5.05	5.70	112.89	0.50	44.00	2.00	6.00	3.00
Dag defect	2.05	0.70	34.14	1.10	4.66	1.56	2.52	2.04

SD, standard deviation; CV, coefficient of variation; Min, minimum value; Max, maximum value, Q1: first quartile, Q3: third quartile, P (50): 50th percentile, P (95): 95th percentile.

**Figure 2:**
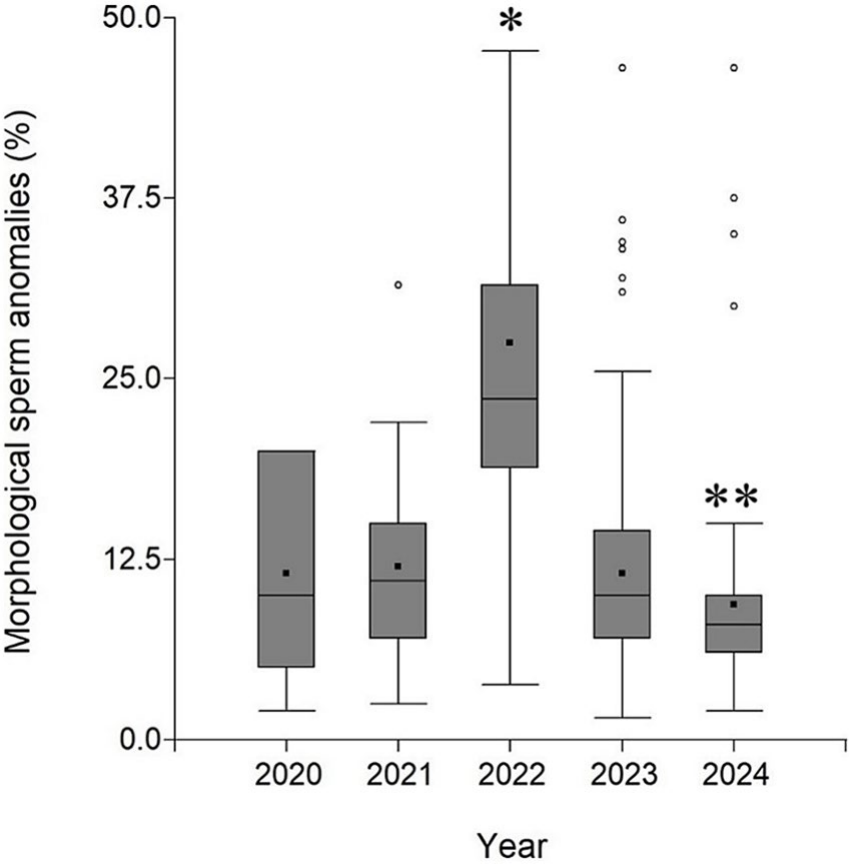
Distribution of sperm morphological anomalies in Brahman bulls from 2020 to 2024 in Costa Rica. Values are mean values (black points), medians (horizontal bars) with 25th and 75th percentiles (boxes); the “whiskers” extend out to the minimum and maximum value. The empty circles correspond to outliers. The asterisk indicates statistical differences between 2022 and the other years. The double asterisk indicates statistical differences between 2024 and other years (*p* < 0.05).

A significant interannual variability was observed in the percentage of sperm morphological anomalies in Brahman cattle, with a notable increase (*p* < 0.05) in 2022. In 2022, the median percentage of sperm anomalies reached its highest value, with greater data dispersion and a high presence of outliers. In contrast, 2020, 2021, 2023, and 2024 exhibited more homogeneous distributions, with lower medians and less dispersion (Figure 2).

## 4 Discussion

Sperm morphology analysis helps to optimize semen dose and predict potential ejaculate quality prior to artificial insemination (42–44). However, different methods are used to assess sperm morphology, such as fixation (typically with formalin) and wet preparations with observations under positive phase contrast or air-dried smears followed by staining and usually bright field microscopy. These variations hinder protocol standardization (45). This high intra and inter laboratory variability has already been reported in several studies (46, 47). In this study, a fixation method without staining was used, applying temperature and pressure to immobilize spermatozoa. This may result in less artifacts (14).

Sperm morphological evaluation is commonly performed using stains such as eosin nigrosin, which allow sperm visualization under bright field microscopy. However, some studies have shown that this methodology may be less precise in detecting sperm morphological anomalies (48, 49) compared to wet preparations using phase contrast microscopy. Live spermatozoa can be immobilized without the need for fixatives by placing them in shallow chambers, which prevents movement and enables more accurate visualization of head and midpiece morphology (50, 51). A key factor in ensuring the reproducibility of sperm morphology evaluation is that the protocol should be simple and involve as few steps as possible, facilitating its uniform application (12, 52, 53). Air drying techniques alter cell morphology (17), whereas wet preparations allow sperm assessment under normal conditions without increasing variability (14, 54).

The overall prevalence of morphologically abnormal sperm in beef bulls was approximately 12% similar to Hanson et al. (55), but lower than other studies (56–58). Although Fitzpatrick et al. (25) have suggested that a normal spermiogram for a bull considered fit and of acceptable fertility should contain fewer than 30% morphologically abnormal spermatozoa. This threshold is general and does not account for specific cutoff values for each type of morphological anomaly, which should be quantified separately due to their differing impacts on fertility (11). Furthermore, sperm morphology analysis remains a valuable tool for assessing bull fertility (23), to the extent that some authors recommend that no more than 20% of sperm in a sample should exhibit head abnormalities (26, 59). Findings from various studies indicate that advanced age is associated with a decline in sperm motility and the percentage of sperm with normal morphology (27, 28, 60), which is different from what was found in the present work. In the present study, the mean values across different age groups, scrotal circumference cohorts, and yearly analyses were below the general threshold. However, as bulls aged, the percentage of sperm morphological anomalies increased, a trend that has also been reported in humans (61, 62).

In humans, as age increases, changes in the testicular parenchyma associated with the aging process occur, affecting both the interstitial space and the germinal compartment, as well as leading to dysfunctional testicular spermiogenesis and sperm maturation (63, 64). In bovine, the scrotal circumference has been associated with the age of the bulls (32, 65), and that could also be associated with mechanisms of metabolic regulation by hormones (66). The present study suggests an association between increased scrotal circumference and sperm morphological anomalies, with a significant increase with a scrotal circumference greater than 47 cm. These factors may help explain the observed effects on sperm motility and morphology (67, 68). Although the human species, compared to other animal species, typically exhibits greater diversity of abnormal sperm forms (69), it is essential to precisely determine the incidence of sperm morphological defects and understand the physiological mechanisms that cause high incidence. Advancing knowledge of the mechanisms involved in spermatogenesis and promoting standardization in sperm morphology evaluation are necessary (62). The use of semen evaluation techniques based on computational systems and the incorporation of artificial intelligence, along with more efficient laboratory procedures, can contribute significantly to improving accuracy and standardization in sperm morphology assessment protocols (70).

During spermiogenesis, the processes of acrosome formation, nuclear reorganization, chromatin condensation, and tail assembly take place (71). Considering that bull spermatogenesis requires approximately 61 days, followed by an epididymal maturation process lasting 11 days (59), most anomalies observed in spermatozoa reflect prior alterations (72) that likely occurred in the seminiferous tubules or, to a lesser extent, in the epididymis. Morphological anomalies in the midpiece and tail are often associated with defects arising in the distal half of the cauda epididymis, leading to spermatozoa with reduced motility or none at all (59), which in turn lowers bull fertility (73–75).

Nuclear vacuolization was the most frequent sperm anomaly, followed by bent tails, loose heads, and distal midpiece reflex. This result is consistent with previous reports on sperm morphological anomalies, except for nuclear vacuoles (58). The anomaly pattern in ejaculates varies both between and within species (76) and can indicate the state of spermiogenesis in the testes and sperm maturation in the epididymis (8). Spermatozoa with abnormal morphology are often correlated with functional alterations during spermatogenesis, such as chromatin condensation defects, acrosomal reaction anomalies, flagellar motility issues, or even increased apoptosis and necrosis (77, 78).

Morphological anomalies also associate with genetic alterations, such as multiple tail defects or headless spermatozoa (79). Proximal cytoplasmic droplets are a spermatogenetic defect (80), and their presence in a high percentage of spermatozoa compromises fertility (81, 82). In the present study, the percentage of proximal cytoplasmic droplets was 2.04%, with minimum and maximum values of 0.47 and 15%, respectively. In bulls, a greater proportion of sperm with proximal droplets in the ejaculate has been negatively correlated with the artificial insemination non return and pregnancy rates (18). Among the head anomalies observed, loose heads were the most common, consistent with previous reports (58). One main cause associated with a high percentage of loose heads is testicular degeneration and sperm senescence resulting from sexual inactivity (59); therefore, sexual status should be considered in animals for reproduction (33).

Certain morphological defects, such as excessive residual cytoplasm and coiled tails, may result from deficient sperm maturation in the epididymis (83). In the case of cytoplasmic droplets, some authors have highlighted their physiological role in sperm hyperactivation, capacitation, and acrosomal reaction (84). Other studies have suggested that excessive residual cytoplasm around the midpiece contains high concentrations of cytoplasmic enzymes, which can lead to increased levels of reactive oxygen species (85); however, incidence of excessive residual cytoplasm has been associated with andrological assessments considered suitable for reproduction (86).

It has been widely documented that an abnormal sperm cell can simultaneously exhibit multiple morphological defects; however, certain combinations occur more frequently than others (62). In the present study, two or more morphological anomalies were occasionally observed together. Other studies have reported that neck abnormalities are more common in sperm with round heads than in those with normal head morphology (87). Similarly, defects in the midpiece, tail, and excessive residual cytoplasm are more frequently detected in sperm with pyriform heads compared to those with normal heads. Some authors suggest that these anomalies may be associated with defective processes during spermiogenesis (76).

In beef cattle, scrotal circumference and semen quality variables such as motility and morphology show a significant correlation with fertility (58, 88). The appearance of morphological abnormalities usually stabilizes in bulls when spermatogenesis normalizes between 14 and 15 months of age, however, this process may take longer in *Bos indicus* breeds (59), which may explain why young bulls’ higher percentages of sperm abnormalities had, but this requires further study. The scrotal circumference is a parameter associated with testicular volume and semen quality (30, 89, 90). Additionally, it has a negative linear correlation with the incidence of primary sperm defects (91). Primary defects or non-compensable defects (*proximal droplets, pyriform heads and diadem defects*) are those that can reach the ovum, block polyspermy, but do not generate a viable embryo, affecting fertility and the performance of the production systems (59).

Also, other external factors such as the season could have an influence on the values of seminal quality in bulls. The study demonstrated a year-on-year variation in the period analyzed. This could be explained because temperatures and weather variations can affect sperm morphology and the condition of animals. Previous studies have shown that the season can cause variations in cell morphology values, both in compensable and non-compensable defects (92). Also, variations in the values of scrotal circumference and cell morphology associated with variations of the season have been demonstrated previously (93, 94).

Sperm morphology evaluation remains a valuable indicator for assessing semen quality in breeding bulls on farms, providing essential information for determining whether a bull is suitable for reproduction, this has been validated by some previous studies (58). It has also been proven that sperm morphology could influence conception rates in bovine and fertility (95). Furthermore, for artificial insemination centers, sperm morphology information helps to optimize higher quality semen doses. The systematic evaluation of sperm morphology should lead to a standardized approach that ensures reproducibility.

## 5 Conclusion

In summary, sperm morphological anomalies in Brahman bulls showed similar incidence regardless of sexual status (breeding or resting), but they varied according to age and scrotal circumference. A higher frequency of anomalies was observed in younger bulls (>2 years) and those with a scrotal circumference greater than 47 cm. Additionally, significant interannual variation was noted, with a notable increase in 2022, followed by a reduction in subsequent years.

The most common morphological defects by anatomical region were loose heads and nuclear vacuoles (head), distal midpiece reflex (midpiece), and bent tails (tail). Among these, the distal midpiece reflex was the most frequent. Continuous evaluation of sperm morphology should be incorporated into genetic selection and reproductive management programs.

Standardization in sperm morphology assessment must ensure reliable results and quality control. Further research is needed to identify specific morphological defects that may impact the success of assisted reproductive techniques, as well as population studies that consider environmental and genetic factors during spermatogenesis to further explore the relationship between sperm morphology, sperm functionality, and reproductive status.

## Data Availability

The original contributions presented in the study are included in the article/Supplementary material, further inquiries can be directed to the corresponding author.

## Footnotes

^1^ https://arriveguidelines.org/
