# Supplementary material for: Effect of age and scrotal circumference on sperm morphology in Brahman bulls using a modified fixation technique
Source: Front Vet Sci. 2025 Aug 8;12:1626425. doi: 10.3389/fvets.2025.1626425 (PMC12371937; doi:10.3389/fvets.2025.1626425)
Supplement: Supplementary file 1 [file Image_1.pdf]

Supplementary Material

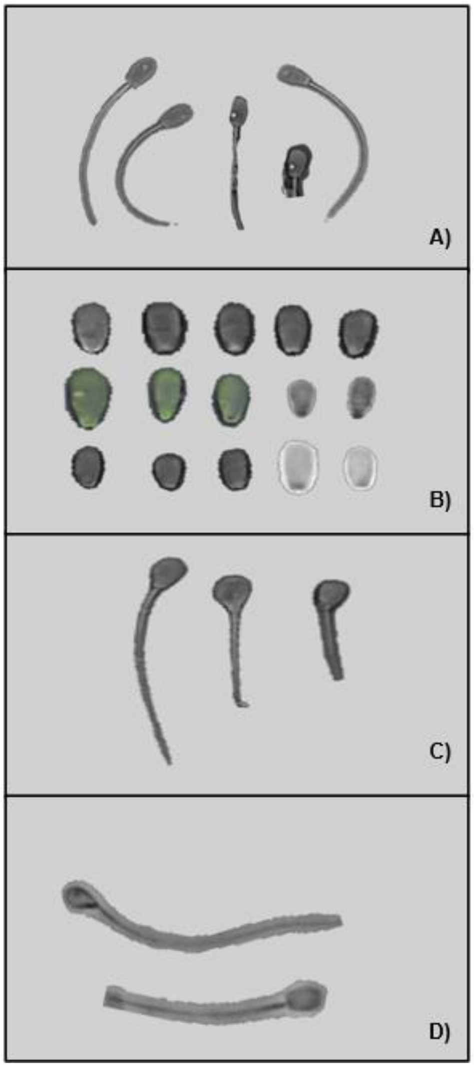

Supplementary Figure 1. Nuclear vacuoles (A), variations of loose head (B), Microcephaly (C) and Pyriform (D) anomalies observed in Brahman bulls' sperm ejaculates using a microscope with phase contrast optics under 400x magnification.
